# Billing Disparities in Home Sleep Testing: The Role of Sleep Medicine Board Certification and Practice Setting

**DOI:** 10.3390/healthcare14132004

**Published:** 2026-07-06

**Authors:** Umesh Ghimire, Heather L. Taylor, Scott R. Houle, Snigdha Pusalavidyasagar, Wajahat Khalil

**Affiliations:** 1 Department of Health Policy & Management, Fairbanks School of Public Health, Indiana University Indianapolis, Indianapolis, IN 46202, USA; 2 Division of Health Policy and Management, School of Public Health, University of Minnesota, Minneapolis, MN 55455, USA; 3 Division of Pulmonary, Allergy, Critical Care and Sleep Medicine, Department of Medicine, University of Minnesota, Minneapolis, MN 55455, USA

**Keywords:** obstructive sleep apnea, home sleep testing, healthcare costs, provider charges, board-certified sleep specialist, OSA diagnosis, health services research

## Abstract

**Highlights:**

**What are the main findings?**

- Board-certified sleep specialists charge less for home sleep tests than non-specialist providers, and this cost difference is consistent across all three types of home sleep test procedures.
- Where and how a provider practices also matters—solo practitioners bill less than hospital-based providers, and fee-for-service payment plans are linked to lower charges than commercial insurance plans.

**What are the implications of the main findings?**

- Sleep apnea diagnostic testing through board-certified sleep specialists and individual practice settings are associated with lower billed charges for home sleep test procedures; however, the actual cost reductions substantially depend on negotiated reimbursement rates, billing structures, and practice setting.
- This study establishes a foundation for future research to evaluate actual insurance reimbursements, patient out-of-pocket costs, billing structures, and practice setting variations. Researchers must investigate these parameters to validate and translate these findings into clinical protocols and healthcare policies aimed at reducing home sleep testing costs.

**Abstract:**

**Background**: The financial burden of diagnostic testing for obstructive sleep apnea (OSA) represents a substantial barrier to treatment initiation, with cost-related access disparities disproportionately affecting the low-income and underinsured population. Home sleep testing (HST) offers a cost-effective diagnostic alternative, yet economic patterns across provider types remain unclear. This study assessed whether board-certified sleep medicine provider (BCSMP) status is associated with differences in provider-billed HST charges and evaluated how organizational and payment contexts influence these charges. **Methods**: A retrospective cross-sectional analysis was conducted using 2019 data from Optum’s de-identified Clinformatics^®^ Data Mart Database (*N* = 61,531 adult HST claims). The main exposure was provider status (BCSMP vs. non-BCSMP). The outcome was total provider-requested charge per HST procedure. Generalized Linear Models with a gamma distribution estimated adjusted charge differences, controlling for organizational context, place of service, and payer type. **Results**: BCSMP encounters had significantly lower adjusted mean HST charges than non-BCSMPs (mean difference: −$78.04; 95% CI: −$89.06 to −$67.02; *p* < 0.001). Individual practitioners charged $168.48 less than hospital-affiliated providers, while group practices and other facilities charged more (all *p* < 0.001). Fee-for-service arrangements were associated with lower charges than commercial and Medicare Advantage plans (*p* < 0.001). **Conclusions**: Board-certified sleep medicine providers and individual practice settings were associated with lower billed charges for home sleep testing; however, these findings do not necessarily reflect actual cost reduction. To translate these baseline charge differences into equitable clinical protocols and healthcare policies, future research must analyze negotiated reimbursement rates, billing structures, and practice environments to determine how these cost parameters impact the overall cost of an OSA diagnosis.

## 1. Introduction

Sleep disorders, encompassing conditions such as sleep apnea, insomnia, and restless legs syndrome, represent a significant public health burden with profound implications for patient well-being and healthcare resource utilization [1,2,3]. Among these, sleep apnea, specifically obstructive sleep apnea (OSA), is remarkably prevalent, affecting an estimated 29.4 million adults in the United States [4]. Despite its prevalence, 80% of adults remain undiagnosed, creating a critical gap in early diagnosis and intervention efforts [4]. Untreated sleep apnea is associated with myriad adverse health outcomes, including increased risk of cardiovascular disease and stroke, and higher healthcare utilization which contributes to substantial direct and indirect costs [5].

The financial burden of diagnostic testing represents a substantial barrier to OSA diagnosis and treatment initiation, with cost-related access disparities disproportionately affecting low-income and underinsured populations [6,7]. The diagnosis of sleep apnea is primarily achieved through sleep tests, with polysomnography (PSG) historically considered the gold standard [8]. However, the resource-intensive nature, high cost, and burden of an overnight laboratory stay associated with PSG have led to the increasing adoption of home sleep testing (HST) as a more accessible and cost-effective diagnostic alternative [9]. HST offers enhanced patient comfort and facilitates data collection in a natural sleep environment, proving beneficial for initial diagnostic pathways [10]. In typical clinical workflow, primary care physicians (PCPs) initially evaluate patients with suspected OSA symptoms and frequently serve as gatekeepers for referrals to specialist care [11]. PCPs may directly order HSTs for initial evaluation or refer patients to board-certified sleep medicine providers (BCSMPs) for specialist assessment [12]. However, billing and procedural responsibility often remains with the ordering or attending provider, creating variations in billing patterns that reflect practice setting and provider specialization rather than purely clinical decision-making [13,14].

Sleep disorder management involves both board-certified sleep medicine providers (BCSMPs) with specialized training in fields like pulmonology, neurology, psychiatry, psychology and sleep medicine, and also non-board-certified sleep medicine providers (non-BCSMPs), who are often generalist family and internal medicine physicians [11]. The three primary CPT codes used for home sleep testing—CPT 95800 (simultaneous recording of heart rate, oxygen saturation, and respiratory analysis), CPT 95801 (minimum monitoring including heart rate, oxygen saturation, and respiratory analysis), and CPT 95806 (comprehensive unattended monitoring with respiratory effort, oxygen saturation, and heart rate)—represent increasing levels of technological complexity and sensor sophistication in HST equipment [15,16]. While the convenience and cost-effectiveness of the HST have positioned it as a cornerstone in OSA diagnosis, there is a gap in the literature when it comes to the cost effectiveness of diagnosis for different providers. Crucially, the interplay between provider characteristics, including specialization and practice setting, and payer category on HST-associated claims remains largely unexamined [13]. This knowledge gap is particularly salient given the prevalence of OSA and the need for health systems to optimize healthcare resource allocation for efficiency and equity in diagnostic and management paradigms [14,17].

This study aims to address this critical gap by describing how provider status, practice settings, and payer type are associated with the costs of home sleep study procedures (CPT codes 95800, 95801, and 95806). We leverage commercial and Medicare Advantage claims data spanning all 50 states to understand how HSTs are utilized for diagnosis, and whether there are any differences in provider-requested charges between BCSMPs and non-BCSMPs. Providers and plan administrators will gain insights into variations in billing practices, especially for common procedures like HSTs. Comprehensive cost analyses such as those presented here offer a compelling foundation for evidence-based policy interventions aimed at enhancing the equitable diagnosis and management of sleep-disordered breathing [18]. Examining the cost differences for HST diagnosis between BCSMPs and non-BCSMPs, to our knowledge, has not been done before. This study contributes to a limited body of literature examining cost variation in HST procedures across provider specialization categories, addressing an important gap in sleep medicine literature.

## 2. Materials and Methods

### 2.1. Study Design and Data Source

We conducted a retrospective cross-sectional analysis utilizing de-identified administrative health claims from the year 2019 within Optum’s de-identified Clinformatics^®^ Data Mart Database [19]. Optum^®^ CDM is a comprehensive database that aggregates medical and pharmacy claims for a large, nationally representative sample of commercial and Medicare Advantage health plan members across all 50 states, with approximately two-thirds of the enrollees in the database covered by commercial plans, and the remaining third covered by Medicare. We focused on data from 2019 to avoid potential confounding influences, atypical healthcare utilization, and cost patterns introduced by the COVID-19 pandemic in 2020 and subsequent years thereafter, consistent with methodology recommendations for administrative claims research [20]. All statistical analyses were performed using Stata/SE 18 (StataCorp LLC, College Station, TX, USA).

### 2.2. Study Cohort

Our initial raw claims dataset included 1,161,460 healthcare encounters with at least one home sleep study procedure. To identify these claims, we retrieved any claim with the following Current Procedural Terminology (CPT) codes: CPT 95800 (HST, unattached equipment, unattended), CPT 95801 (HST, unattached equipment, unattended), and CPT 95806 (HST, unattended, with respiratory effort, oxygen saturation, and heart rate).

Inclusion criteria: We identified adults (aged 18 years or older) with continuous enrollment in the health plan for at least 12 months prior to the index HST date, to ensure the capture of all claims from the HST index date. Using this cohort who had received a HST procedure, we verified that they had a subsequent obstructive sleep apnea diagnosis (ICD-10 code G47.33) in 2019. Exclusion criteria: Claims where HST was combined with other billable events on the same date of service (*N* = 70,274) were excluded, as these co-occurring procedures would inflate billed charges and prevent attribution to HST specifically. Duplicate records for the same patient on the same date of service were not included in the analysis, retaining only one record per date. The final analytical sample consisted of 61,531 claims.

### 2.3. Outcome

Our primary dependent variable for this study was the total provider-requested charges (i.e., billed amounts) for each HST procedure prescribed. This study examines provider-billed charges, not actual insurance payments, patient out-of-pocket costs, or clinical outcomes. Billed charges represent the amount providers submit to insurers and do not reflect negotiated rates, reimbursement amounts, or what patients ultimately pay. To minimize the influence of extreme values common in healthcare cost data, all billed charges were Winsorized at the 1st and 99th percentiles. Specifically, charges falling below the 1st percentile were replaced with the 1st percentile value, and values exceeding the 99th percentile were replaced with the 99th percentile value. This approach was selected because it mitigates the impact of outliers without removing them entirely, preserving the distribution’s tails [21]. Winsorization was used as a standard practice in health economics research and has been validated for healthcare cost data with similar distribution characteristics [22]. Sensitivity analyses conducted during preliminary data exploration confirmed that Winsorization thresholds at the 5th/95th percentiles yielded similar results and did not materially alter the main conclusions regarding charge differences.

### 2.4. Independent Variables

Our key explanatory variable of interest was a binary indicator of provider board certification status: board-certified sleep medicine provider (BCSMP) versus non-BCSMP (generalist family or internal medicine physician, or physician assistant/nurse practitioner in primary care settings). Rather than restricting the non-BCSMP category to primary care physicians alone, we employed a taxonomy-based classification approach that captures the full spectrum of non-sleep-medicine providers in claims data, including family medicine and internal medicine practitioners, physician assistants, nurse practitioners, and other specialists (e.g., cardiologists, pulmonologists, equipment technicians) who do not hold board certification in sleep medicine. We utilized the National Uniform Claim Committee (NUCC) Taxonomy, a standardized nomenclature system that catalogs medical specialties with corresponding codes across all clinical domains [23]. BCSMPs were identified using ten discrete NUCC sleep medicine specialty codes (207QS1201X, 207RS0012X, 207YS0012X, 2080S0012X, 2084S0012X, 207RM1200X, 207N0300X, 207RP1001X, 207RR0500X, 207RS0010X), matched against both primary and secondary taxonomy fields within the Optum^®^ CDM dataset [24]. All remaining providers, regardless of clinical specialty or credential type, were conservatively categorized as non-BCSMPs. This deterministic approach ensures operational transparency by defining non-BCSMPs through explicit absence of sleep-medicine-specific taxonomy assignment rather than restrictive exclusion criteria, thereby encompassing the heterogeneous provider types that bill for HST services in contemporary practice [25].

We also included several categorical covariates, including the organizational context of care delivery for each provider (individual practice, hospital-affiliated, group practice, or other facility) [26]. The place of service represents the setting where procedures were rendered (e.g., home and residential care, clinical office, institutional care, or other). Place of service was included in the analysis to capture whether HSTs were administered in settings optimized for diagnostic testing (offices, clinics) versus institutional settings, which may incur additional facility fees. These variables were selected because organizational setting has been documented as a significant predictor of healthcare costs and billing practices in prior research [27]. Controlling for payer type is standard in healthcare cost analyses; the analysis includes the administrative context of the payer, categorizing coverage into commercial and Medicare Advantage plans, capitation plans, or fee-for-service. Finally, to account for unobserved state-specific factors that could influence healthcare costs (i.e., variations in state-level regulations, market structures, regional practice patterns, and provider density), we included the US Census division indicator for each provider’s practice location.

### 2.5. Analysis

Our analytical plan involves two main stages. We first calculated frequencies and column percentages for all variable characteristics stratified by BCSMP and non-BCSMP classification. Pearson’s chi-squared tests were used to assess significant distributional differences in charge between BCSMP and non-BCSMP for each encounter (payer) and provider characteristic. Second, to analyze the adjusted association between provider BCSMP status and total provider-requested HST charges, we utilized Generalized Linear Models (GLMs). Given the skewed, non-normal nature of healthcare cost data, we employed GLMs with a gamma distribution and a log link function, as recommended in the health economics literature [28]. This specification is more appropriate than ordinary least squares regression because it accommodates the skewness and heteroscedasticity inherent in healthcare costs and ensures that predicted values remain positive, allowing for a proportional interpretation of results [28]. Raw coefficients from the log-linked GLMs were exponentiated to derive adjusted cost ratios; however, for interpretability, we report adjusted marginal effects (absolute dollar differences) [29]. These marginal effects represent the average predicted difference in charges for HST procedures associated with each variable, holding other covariates constant at their means. All models were adjusted for organizational context, place of service, and U.S. Census division. Statistical significance was set at *p* < 0.05.

## 3. Results

Of the 61,531 claims included in this analysis, HST procedures were provided by 24,444 (39.73%) BCSMPs and 37,087 (60.27%) non-BCSMPs (Table 1). The vast majority of BCSMPs practiced in individual settings (97.67%), whereas non-BCSMPs practiced in a variety of settings (individual (31.09%), hospital (24.62%), group practice (20.58%), and other facility (23.71%) settings). While both groups predominantly engaged in non-encounter/administrative activities, BCSMPs showed a slightly higher proportion of capitated (1.18% vs. 0.87%, *p* < 0.001) and fee-for-service (1.81% vs. 1.22%, *p* < 0.001) encounters. Geographic distribution varied significantly; however, both non-BCSMPs (38.61%) and BCSMPs (24.42%) showed the highest concentrations in the South Atlantic, with secondary concentrations in the West South Central and East North Central regions.

Non-BCSMPs were more likely than BCSMPs to bill for CPT 95800 (27.57% vs. 16.82%, *p* < 0.001), while BCSMPs more frequently utilized CPT 95806 (82.89% vs. 72.16%, *p* < 0.001). CPT 95801 showed no statistically significant difference in distribution between provider types (*p* = 0.543). CPT 95801 accounted for only 169 claims (0.28%) of the total 61,531 claims—substantially fewer than CPT 95800 (*N* = 16,324; 26.53%) and CPT 95806 (*N* = 44,820; 72.86%). Consequently, the CPT 95801 findings must be interpreted with caution, as this limited sample size restricts statistical power and may obscure meaningful variations in provider behavior.

Table 2 presents an analysis of two-sample *t*-tests with equal variances to compare the differences in mean charges for procedure codes 95800, 95801, and 95806 when services are rendered by BCSMPs compared to non-BCSMPs. For HST (CPT 95800), non-BCSMPs billed significantly higher than BCSMPs. Similarly, for CPT 95801, despite the small sample size, non-BCSMPs’ average charge remained significantly higher than BCSMP charge. For home sleep study—respiratory effort procedures (CPT 95806), the most commonly used HST procedure, non-BCSMPs charged substantially higher amounts, representing an absolute difference of $343.71 or 97.8% higher charges than BCSMPs.

**Figure 1:**
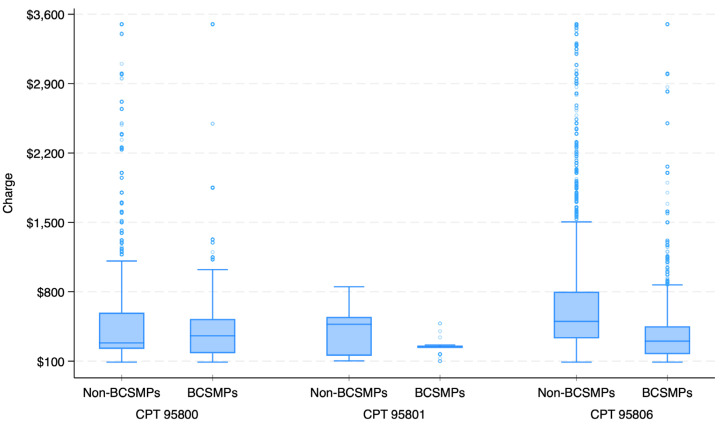
Distribution of charges by BCSMPs ^a^ versus Non-BCSMPs ^b^ and CPT ^c^ codes for home sleep test procedures. ^a^ BCSMPs: Board-certified sleep specialist providers; ^b^ Non-BCSMPs: Non-Board-certified sleep specialist providers; ^c^ CPT: Current Procedural Terminology; CPT 95800: HST, unattached equipment, unattended; CPT 95801: HST, unattached equipment, unattended; CPT 95806: HST, unattended, with respiratory effort, oxygen saturation, and heart rate. Charges are in 2019 United States Dollars.

Figure 1 shows the distribution of charges for home sleep test CPT codes by provider specialization, comparing non-BCSMPs and BCSMPs. For CPT 95800 and 95806, non-BCSMPs demonstrate higher median charges and a wider interquartile range compared to BCSMPs. The median charge for CPT 95806 by non-BCSMPs is higher than that for BCSMPs, with non-BCSMPs also exhibiting a greater number of outliers at higher charge levels. The lowest cost difference was associated with CPT 95801, although non-BCSMPs still charge at higher rates for these procedures than BSCMPs. Overall, BCSMPs exhibit a more concentrated distribution of lower charges across all three CPT codes.

Table 3 presents the adjusted estimated mean differences in total provider-requested charges for HST procedures, derived from post-estimation marginal effects calculations following GLM estimation. Across all HST procedures combined, BCSMPs charged significantly less than non-BCSMPs (−$78.04 (95% CI: −$89.06, −$67.02)). This represents a 13.7% reduction in charges for BCSMP-ordered or -provided HSTs relative to the non-BCSMP mean charge of $569.97. Provider organizational context also demonstrated substantial charge differentials relative to group practices. Compared to hospital-affiliated providers (reference category), individual practitioners were associated with $168.48 (95% CI: −$183.08, −$153.88) lower charges for HST procedures. Conversely, group practices ($313.27 higher; 95% CI: $297.10 to $329.43) and other facilities ($224.26 higher; 95% CI: $208.83 to $239.70) were associated with significantly higher charges, potentially reflecting administrative costs, coordination fees, or greater utilization of ancillary services. Conversely, group practices ($313.27 higher; 95% CI: $297.10, $329.43) and other facilities ($224.26; 95% CI: $208.83, $239.70) were associated with significantly higher charges for HST procedures than hospital-based providers. Regarding payment context, both capitated encounters and fee-for-service payments were associated with lower charges compared to commercial and MA plan categories. Fee-for-service arrangements were associated with the lowest charges (-$160.85; 95% CI: −$195.05, −$126.65, *p* < 0.001).

## 4. Discussion

This study examined the associations between provider and encounter characteristics and home sleep study procedure charges using administrative claims data from the United States. Our findings demonstrate that billed charges for HST procedures differ significantly across provider specialization, organizational setting, and payment mechanism. Our findings showed that BCSMPs are associated with lower billed charges compared to non-BCSMPs; however, this association must be interpreted in the context of substantial practice setting differences between groups and potential confounding mechanisms discussed below.

This study contributes to a limited body of literature examining charge variation in HST procedures, addressing a gap in sleep medicine research [30]. Overall, across all HST procedures (95800, 95801, 95806), BCSMPs exhibited lower billed charges compared to non-BCSMP. These three CPT codes represent the common options for diagnosing OSA via HST, varying in their complexity and the level of monitoring provided [5,15]. CPT 95801, typically a simpler unattended study, generally involves fewer sensors and is considered the least complex, which is reflected in its overall lower mean charges for both provider types. Non-BCSMP mean charges for CPT 95801 were substantially greater than those of BCSMPs. Despite its low operational complexity, the minimal utilization and correspondingly small sample size for CPT 95801 suggest it is less preferred among providers or less commonly billed in the Optum^®^ CDM population, explaining the lack of a statistically significant difference. Further investigation into CPT 95801 billing trends would provide valuable insight into these utilization patterns. CPT 95800, an unattended home sleep study, involves a higher degree of professional oversight, contributing to its increased complexity [15]. CPT 95806, which includes respiratory airflow, oxygen saturation, heart rate, and respiratory effort, exhibited the largest absolute difference in mean charges across provider types. The large charge differential may reflect the distinct billing structures and comprehensiveness service scope inherent to CPT 95806 [30]. BCSMPs can provide comprehensive HST evaluation, directed therapeutic recommendations including continuous positive airway pressure (CPAP) prescriptions, assessment of co-morbid sleep disorders (e.g., insomnia), and additional diagnostic testing without requiring external specialist consultation. In contrast, non-BCSMPs may need to refer complex cases to BCSMPs for interpretation, therapy direction, and comorbidity assessment, resulting in additional consultation fees and diagnostic charges, thereby increasing total billed amounts [24].

Our findings reveal that BCSMPs charge lower fees across all HST procedures, a variance that could be attributable to several mechanisms. First, it is possible that this difference may stem from the enhanced training and expertise of BCSMPs in sleep medicine, which could lead to greater cost efficiencies in identifying and analyzing the severity of OSA. The inherent value of specialty board certification in sleep medicine as a predictor of high-quality and cost-effective healthcare is increasingly recognized. A growing body of research indicates a generally positive association between board certification and clinical outcomes, including higher satisfaction rates [31], reduced time to consultation [32], and greater adherence to positive airway pressure therapy [33]. While assessing treatment cost was outside the scope of this study, the precision of BCSMP-led diagnosis is critical for effective management [34]. Second, this pattern may be partially explained by billing and referral structures not fully captured by claim taxonomy [24]. BCSMPs, with their advanced training and resources, are equipped to manage the entire spectrum of sleep disorders [33]. In contrast, non-BCSMPs, while capable of prescribing diagnostic tests like HSTs, may lack explicit training in sleep or chronobiologic illnesses [35]. Consequently, they may need to outsource BCSMPs for interpretation and subsequently bill a global or bundled code. Given that two thirds of the Optum^®^ CDM dataset comprised commercial employer-sponsored health plan claims, it is possible that the observed difference between non-BCSMP and BCSMP charges is due to bundled consultation with an outside BCSMP that encompasses both the technical HST component and the professional interpretation component. While the Centers for Medicare and Medicaid Services (CMS) have strict guidelines against bundling [36], commercial insurers face no such restrictions against PCPs contracting with BCSMPs to bill the full global code for diagnosis and interpretation [36,37]. However, CPAP prescription regulations vary widely by state; some allow credentialed PCPs to initiate therapy via established clinical protocols [38]. Similarly, commercial insurance plans and Medicare may have differing policies regarding CPAP prescription authority and coverage [17]. Third, non-BCSMPs may be more likely to utilize facility-based infrastructure, higher-acuity settings, or ancillary services such as technical scoring, device activation, or dispensing fees when conducting more complex HSTs, thereby increasing total billed charges [39]. Future research should explore the specific billing rules and contractual language utilized by commercial insurance providers.

The heterogeneity in charge differences between BCSMPs and non-BCSMPs for similar services suggests inefficiencies within the healthcare system, which may be directly tied to varying overhead costs or provider billing discretion [40]. Because the non-BCSMPs cannot independently prescribe CPAP therapy without specialist consultation, this additional specialist referral step within the care process could possibly delay the treatment [41]. While delays in CPAP initiation are plausible in some clinical contexts, the assumption that non-BCSMP involvement systematically delays treatment requires empirical validation within specific regulatory and insurance contexts. Without access to granular data on specific billable components (technical vs. professional components, ancillary services), the precise drivers of this variability remain speculative.

Beyond specialty, provider work setting, particularly, office- or clinic-based settings were associated with significantly lower charges compared to hospital-affiliated providers and other facilities. In general, HSTs reduce the need for sleep-lab facility charges in OSA screening [42]. However, complex sleep disorders such as central sleep apnea, narcolepsy, parasomnias, seizures, or movement disorders, require more extensive tests [43,44,45]. This large and statistically significant difference suggests that institutional overhead and facility charges, such as a device activation fee, a dispensing fee, a technical scoring fee, or all of the above, require more extensive testing [40]. Another explanation for this substantial difference in charges is that practice setting may be a major confounder, as organizational context strongly influences billed charges [26]. Specifically, it is difficult to fully differentiate whether lower BCSMP charges are attributable to sleep medicine board certification itself, or to the fact that BCSMPs were predominantly practicing in lower-cost individual settings [42]. Although our GLMs are statistically adjusted for organizational context, residual confounding is likely to remain [46]. Therefore, we recommend future research explore charge patterns by comparing individual practices versus hospital-based settings separately to clarify whether the BCSMP advantage persists independently of organizational context.

Furthermore, payment mechanisms influenced charge, with both capitated and fee-for-service plans demonstrating lower charges than commercial and MA plans. Capitation often incentivizes efficient resource use due to fixed payments per enrollee, while fee-for-service, when applied to streamlined diagnostic services like HSTs, can reflect direct service costs without additional administrative charges [16]. Given that home sleep testing has been shown to be more cost-effective than in-laboratory polysomnography and is associated with reduced 30-day hospital readmission and emergency department visit rate [47], promoting efficient delivery of HSTs is a logical first step to OSA diagnosis.

This study possesses several strengths and limitations. As a strength, this study is the first to examine cost differences in HST procedures across BCSMPs and non-BCSMPs. In addition, we conducted our analysis using a nationally representative sample of administrative health claims from Optum^®^ CDM enhancing generalizability of our findings. However, several limitations warrant consideration. First, as a retrospective cross-sectional study, we cannot infer causality, nor account for all potential confounding factors that may be related to the observed cost differences. Second, we acknowledge that claims data lack granular clinical information including OSA severity measures. Although OSA severity itself may not directly influence HST technical charges, differences in patient complexity between BCSMP and non-BCSMP cohorts could influence testing patterns and billing practices [24]. However, if more complex patients (those with obesity, COPD, heart failure, or multiple comorbidities) are referred to hospital-based or facility-based settings and non-BCSMPs, this could create residual confounding wherein organizational setting may account for higher non-BCSMP charges. Furthermore, while claims data are reliable for billing and diagnoses, they lack granular demographic, socioeconomic, and clinical factors, all of which could further contextualize the encounters. Third, the Optum^®^ CDM database comprises approximately two-thirds commercial employer-sponsored plan members and one-third Medicare Advantage enrollees, but explicitly excludes patients covered by traditional (non-Advantage) Medicare and Medicaid programs. These excluded populations represent substantial portions of the U.S. insured population; notably, their payment structures differ fundamentally from the commercial and Medicare Advantage plans represented in this data. Fourth, although we utilized comprehensive provider specialty codes, administrative claims data may not consistently capture all provider specialties, especially secondary or tertiary ones, across all claims. This can occur due to billing system prioritization of primary service taxonomies or variations in reporting requirements across different payers or encounter types [48]. Nevertheless, we used the Optum^®^ CDM taxonomy listing and cross-referenced with the Inovalon taxonomy listing to increase the precision of identifying each provider’s specialty. Finally, we acknowledge that the field of HST underwent a significant technological and structural transformation following the expansion of telehealth policies during the COVID-19 pandemic [49,50]. However, because our underlying claims database is from 2019, this study focuses strictly on baseline diagnostic CPT coding and billing variations. Consequently, we excluded longitudinal treatment pathways, such as remote CPAP titration and digital adherence monitoring via telehealth, which fell outside the operational scope of this analysis. Future research using post-pandemic data is needed to evaluate how ongoing virtual health care delivery models systematically alter both diagnostic coding precision and overall care-episode costs.

## 5. Conclusions

This study offers valuable insights into the factors associated with HST charges. Our findings demonstrate statistically significant associations between provider board certification, practice organizational setting, and HST charges; however, these associations do not necessarily reflect causal relationships or imply superior clinical quality of BCSMP-provided services. BCSMPs and individual practice settings are linked to lower HST charges, while non-BCSMPs, hospital-affiliated providers, and other facilities are associated with higher charges. Future research incorporating clinical outcomes, real-world payment data, and patient-centered measures is essential for translating these charge reduction findings into policy recommendations that enhance both the efficiency and equitable access to OSA diagnosis.

## Figures and Tables

**Table 1:** Claim characteristics reported in all 2019 home sleep test claims and by provider type (Non-BCSMPs ^a^ versus BCSMPs ^b^) (*N* = 61,531).

Characteristic	Total	Non-BCSMPs	BCSMPs	*p*-Value *
**Provider type**				<0.001
Individual	35,404 (57.54)	11,530 (31.09)	23,874 (97.67)	
Hospital	9155 (14.88)	9130 (24.62)	25 (0.1)	
Group practice	8026 (13.04)	7634 (20.58)	392 (1.6)	
Other facility	8946 (14.54)	8793 (23.71)	153 (0.63)	
**Payer category**				<0.001
Commercial & Medicare Advantage Plans	60,026 (97.55)	36,314 (97.92)	23,712 (97.01)	
Capitation Plans	610 (0.99)	321 (0.87)	289 (1.18)	
Fee-for-Service	895 (1.45)	452 (1.22)	443 (1.81)	
**Division**				<0.001
New England	1418 (2.3)	646 (1.74)	772 (3.16)	
Middle Atlantic	3391 (5.51)	1441 (3.89)	1950 (7.98)	
East North Central	7672 (12.47)	4442 (11.98)	3230 (13.21)	
West North Central	5417 (8.8)	2875 (7.75)	2542 (10.4)	
South Atlantic	20,290 (32.98)	14,320 (38.61)	5970 (24.42)	
East South Central	2965 (4.82)	1464 (3.95)	1501 (6.14)	
West South Central	9282 (15.09)	5662 (15.27)	3620 (14.81)	
Mountain	5619 (9.13)	3076 (8.29)	2543 (10.4)	
Pacific	5477 (8.9)	3161 (8.52)	2316 (9.47)	
**CPT ^c^ procedures billed**				
CPT 95800	14,337 (23.3)	10,226 (27.57)	4111 (16.82)	<0.001
CPT 95801	169 (0.27)	98 (0.26)	71 (0.29)	0.543
CPT 95806	47,025 (76.42)	26,763 (72.16)	20,262 (82.89)	<0.001
**Total home sleep tests**	61,531 (100.00)	37,087 (100.00)	24,444 (100.00)	

^a^ Non-BCSMPs: Non-board-certified sleep specialist providers; ^b^ BCSMPs: Board-certified sleep specialist providers; ^c^ CPT: Current Procedural Terminology; CPT 95800: Sleep study, unattended, simultaneous recording of heart rate, oxygen saturation, respiratory analysis (e.g., quantitative waveform of respiratory movement or airflow), and sleep time; CPT 95801: Sleep study, unattended, simultaneous recording; minimum of heart rate, oxygen saturation, and respiratory analysis (e.g., quantitative waveform of respiratory movement or airflow); CPT 95806: HST, unattended, with respiratory effort, oxygen saturation, and heart rate. * A Pearson chi-square test of independence was performed to assess the association between provider type and the inclusion of respiratory effort data. Percentages represent the proportions of the column. The *p*-value indicates the statistical significance of the association, with *p*-value < 0.05 indicating statistical significance.

**Table 2:** Mean charges for home sleep test (HST) procedures by provider specialty status (non-BCSMPs ^a^ versus BCSMPs ^b^).

HST Procedure	Provider Specialty	*N*	Mean Charge ^§^	95% Confidence Interval	Difference in Means (Non-BCSMP—BCSMP)
CPT ^c^ 95800					73.70 *
	Non-BCSMP	10,226	571.29	[558.01, 584.57]	
	BCSMP	4111	497.59	[479.50, 515.68]	
CPT 95801					155.79 *
	Non-BCSMP	98	392.77	[350.71, 434.84]	
	BCSMP	71	236.98	[221.70, 252.26]	
CPT 95806					343.71 *
	Non-BCSMP	26,763	694.49	[686.82, 702.16]	
	BCSMP	20,262	350.78	[346.91, 354.64]	
Total HSTs ^d^					284.58 *
	Non-BCSMP	37,087	659.72	[653.06, 666.39]	
	BCSMP	24,444	375.14	[370.67, 379.61]	

^a^ Non-BCSMP: Non-board-certified sleep specialist provider; ^b^ BCSMP: Board-certified sleep specialist provider; ^c^ CPT: Current Procedural Terminology; ^d^ HST: home sleep test; CPT 95800: Sleep study, unattended, simultaneous recording of heart rate, oxygen saturation, respiratory analysis (e.g., quantitative waveform of respiratory movement or airflow), and sleep time; CPT 95801: Sleep study, unattended, simultaneous recording; minimum of heart rate, oxygen saturation, and respiratory analysis (e.g., quantitative waveform of respiratory movement or airflow); CPT 95806: HST, unattended, with respiratory effort, oxygen saturation, and heart rate. * The difference in mean charges is statistically significant at *p* < 0.05 for all HST procedures ^§^ Unadjusted charges in 2019 United States Dollars.

**Table 3:** Adjusted generalized linear model coefficients for charges for home sleep test procedures.

Variable	Coefficient ($)	95% Confidence Interval	*p*-Value
**Provider Type**			
(Ref: Non-BCSMP ^a^)			
BCSMP ^b^	−78.04	[−89.06, −67.02]	<0.001
**Provider Setting**			
(Ref: Hospital)			
Individual	−168.48	[−183.08, −153.88]	<0.001
Group practice	313.27	[297.10, 329.43]	<0.001
Other facility	224.26	[208.83, 239.70]	<0.001
**Payer Category**			
(Ref: Commercial & MA Plans)			
Capitation Plans	−43.33	[−84.29, −2.37]	0.038
Fee-for-Service	−160.85	[−195.05, −126.65]	<0.001

^a^ Non-BCSMPs: Non-Board-certified sleep specialist providers; ^b^ BCSMPs: Board-certified sleep specialist providers.

## Data Availability

The dataset supporting the conclusions of this study, Optum’s de-identified Clinformatics^®^ Data Mart Database, is a proprietary administrative health claims database. Individual patient-level data are not publicly available and cannot be shared, as access is governed by a data use license agreement between the data owner (Optum) and the investigators. Data requests may be submitted directly to Optum^®^ CDM (https://www.optum.com/solutions/data-analytics, accessed on 1 May 2024).

## Abbreviations

The following abbreviations are used in this manuscript:

BCSMP	Board-certified sleep specialist provider
CMS	Centers for Medicare and Medicaid Services
CPAP	Continuous Positive Airway Pressure
CPT	Current Procedural Terminology
HST	Home Sleep Test
ICD	International Classification of Diseases
Non-BCSMP	Non-Board-certified sleep specialist provider
MA	Medicare Advantage
OSA	Obstructive Sleep Apnea
PCPs	Primary Care Providers
